# Using Pharmacogenomic Databases for Discovering Patient-Target Genes and Small Molecule Candidates to Cancer Therapy

**DOI:** 10.3389/fphar.2016.00312

**Published:** 2016-09-29

**Authors:** José E. Belizário, Beatriz A. Sangiuliano, Marcela Perez-Sosa, Jennifer M. Neyra, Dayson F. Moreira

**Affiliations:** ^1^Department of Pharmacology, Institute of Biomedical Sciences, University of São PauloSão Paulo, Brazil

**Keywords:** cancer genome, CellMiner, Connectivity Map, cBioPortal, pharmacogenomics

## Abstract

With multiple omics strategies being applied to several cancer genomics projects, researchers have the opportunity to develop a rational planning of targeted cancer therapy. The investigation of such numerous and diverse pharmacogenomic datasets is a complex task. It requires biological knowledge and skills on a set of tools to accurately predict signaling network and clinical outcomes. Herein, we describe Web-based *in silico* approaches user friendly for exploring integrative studies on cancer biology and pharmacogenomics. We briefly explain how to submit a query to cancer genome databases to predict which genes are significantly altered across several types of cancers using CBioPortal. Moreover, we describe how to identify clinically available drugs and potential small molecules for gene targeting using CellMiner. We also show how to generate a gene signature and compare gene expression profiles to investigate the complex biology behind drug response using Connectivity Map. Furthermore, we discuss on-going challenges, limitations and new directions to integrate molecular, biological and epidemiological information from oncogenomics platforms to create hypothesis-driven projects. Finally, we discuss the use of Patient-Derived Xenografts models (PDXs) for drug profiling *in vivo* assay. These platforms and approaches are a rational way to predict patient-targeted therapy response and to develop clinically relevant small molecules drugs.

## Introduction

In last decade, a number of high-throughput large scale cancer genomic technologies have generated very comprehensive and complex datasets (Cline et al., 2013; Robbins et al., 2013; Van Allen et al., 2013; Friedman et al., 2015; Ledford, 2015). Some of key features of each type of tumor and individual patient’s cancer including a specific pattern of DNA mutations, deletion, amplification, gene rearrangements, translocation, microsatellite instability, and epigenetic alterations have been revealed, but not fully understood (Hanahan and Weinberg, 2011; Cline et al., 2013; Robbins et al., 2013; The Cancer Genome Atlas Research et al., 2013; Van Allen et al., 2013; Lawrence et al., 2014; Forbes et al., 2015). Numerous web-based oncogenomic portals for assessment of tumor genetic profiling derived from a very large number of patients have been served as powerful tools for discovery and implementation of personalized cancer medicine (Ciriello et al., 2013; Van Allen et al., 2013; Friedman et al., 2015; Klonowska et al., 2015). The use of genomic information to identify and develop innovative therapies depends on development of statistical, mathematical and computational methods. Translational bioinformatics is an emerging field dedicated on applying informatics to find genomic alterations that can be used to development of precision medicine strategies using multi-omics datasets (Ciriello et al., 2013; Dienstmann et al., 2015; Friedman et al., 2015). There are several large-scale cancer genomics platforms available for cancer researchers querying molecular profiles and clinical drug response from experimental and clinical trial studies (Van Allen et al., 2013; Dienstmann et al., 2015). These rich data sets allow clinical applications of genomic markers derived from treatment response and/or adverse events into biologic insights and new treatments. Familiarity with bioinformatics tools is one of present challenges to explore genomics projects aiming to reveal cancers’ gene drivers and thereby planning a rational sequence of targeted cancer therapies.

Further comprehensive histological and molecular characterization of various types of cancer cells depends on systems biology strategies to measure, create models and manipulate appropriated tissue cell culture, animal models and patient cohorts (Floor et al., 2012; The Cancer Genome Atlas Research et al., 2013; Belizário et al., 2015). Initially, system-level approaches have revealed a number of genetic and epigenetic alterations that contribute to oncogenesis, progression and metastasis of cancer cells (Hanahan and Weinberg, 2011). Recent studies on tumor pathobiology in hematological and solid cancers have revealed that heterogeneity is the major cause of poor drug efficacy and response duration, within and between individual patient groups (Visvader, 2011; McGranahan and Swanton, 2015). Because of heterogeneity at cellular, molecular pathways and pathophysiological levels, cancer cells that respond to particularly therapeutic treatment can rapidly adapt and develop extrinsic and intrinsic resistance changing their driver mutation signaling pathways (Luo et al., 2009; Carragher et al., 2012; McGranahan and Swanton, 2015). The targeted inhibition of a unique protein (e.g., protein kinase) in a driver cancer pathway many times results in the activation of pre-existing genetic alterations in tumor cell clones (Nijhawan et al., 2012; Holohan et al., 2013). Thus, the dynamic of variations in cancer pharmacogenomics is much more complex and require identification of target-shifting players, such as molecules and pathways at a certain time point along with the treatment (Bozic et al., 2013; Dienstmann et al., 2015; Xia et al., 2014). Therefore, only a high-resolution and broader view of signaling systems of the cancer genome could allow us to understanding the complex mechanisms that make tumor cells to subvert single- and multi-agent therapies.

In 1946, Louis Goodman and Alfred Gilman Goodman at Yale University were the first researchers to explore a synthetic molecule called “synthetic lymphocidal chemical” in a patient (J.D) with massive lymphoma. The patient presented partial response to the treatment. Afterward, the nitrogen mustard molecules were developed as chemical alkylating agents to treat human patients by Haddow et al. (1948). Until today, the screening of chemicals and natural products derived from microbial and plant species have been a preferential route to discover new candidates for cancer therapy (Neidle and Thurston, 2005; Carragher et al., 2012). With advances in the protein purification and crystallography methodologies, the so-called *in silico* approach for predicting and designing ligands to target structure using large virtual libraries have also been used (Neidle and Thurston, 2005). Research Collaboratory Structural Bioinformatics Protein Data Bank (RCSB PDB database^1^) and PDBbind database^2^ are examples of repositories of proteins structures, nucleic acids, and complex assemblies for *in silico* experiments (Neidle and Thurston, 2005). The open-source target validation programs for large scale protein kinase inhibitor screening have been a fruitful way for sharing knowledge and reagents to understanding protein kinases signaling pathways and drug discovery (Edwards et al., 2015; Campbell et al., 2016; Elkins et al., 2016). The development of highly selective ATP-competitive inhibitors for intracellular and membrane tyrosine and serine-threonine kinases (RTKs) has had a great impact in cancer therapy. For instance, Imatinib for the Abelson kinase (ABL), Lapatinib for epidermal growth factor receptor (EGFR) and ERBB2 transmembrane protein kinases, and novel inhibitors such as Vemurafenib to mutated BRAF^V 600E^ protein kinase (Elkins et al., 2016). However, the extensive redundancy of RTK-transducing pathways, cross-reactivity, toxicity and tumor resistance remain major challenges and limitations of targeting key protein kinases for cancer treatment (Fabbro et al., 2015). Currently, 33 protein kinase inhibitors approved by Food and Drug Administration (FDA) are available for patient clinical treatment (Fabbro et al., 2015).

*In vitro* and *in vivo* assays based on cancer cell lines, or tumor xenograft in immunodeficient mice, are often used to high-throughput screening and to assess the cytotoxic effects of small molecules (Sharma et al., 2010). Various morphological and biochemical methods are usually used as read-out for therapeutic efficacy of compounds targeting cell death modulators of apoptosis, necroptosis, pyroptosis, ferroptosis, and autophagy in cancer cell lines (Wolpaw et al., 2011; Dixon et al., 2012; Wolpaw and Stockwell, 2014). More recently, new *in vivo* mouse model named Patient-Derived Xenografts model (PDX), or avatar, has been developed to validate the activity and efficacy of novel agents for systemic therapies (Hidalgo et al., 2014). NOD/*scid*/IL2*rg*^null^ (NSG) mice, which lack T, B and NK cells, have been used to generate collections of human tumor PDX models (Hidalgo et al., 2014; Shultz et al., 2014). PDXs form well-organized three-dimensional architecture and display phenotypic and functional heterogeneity of tumors as well as cancer stem cells (CSC) repopulation (Chaffer et al., 2011; Gupta et al., 2011; Williams et al., 2013). This system also provides immune cells and non-malignant components of stromal microenvironment that have direct implications in CSC’s escape from immune surveillance (Chaffer et al., 2011; Gupta et al., 2011; Östman and Pietras, 2013; Zitvogel et al., 2013) as well as metastasis (Chaffer and Weinberg, 2011). There is convincing data supporting can stem cell origin and their possible *de novo* rise from non-CSCs *in vitro* and *in vivo* (Chaffer et al., 2011; Gupta et al., 2011). Conditionally reprogramming of epithelial cells (CRC) induced by a Rho kinase inhibitor (Y-27632), in combination with fibroblast feeder cells, is a cell culture technique that enable the growth and establishment of rare cancer cell lines, including CSCs, from animal and patient’s small biopsy (Liu et al., 2012; Gach et al., 2013). These new systems will greatly accelerate the identification of new biomarkers for cancer progression and small molecules candidates to cancer therapy.

Considerable effort has been put in system pharmacology approaches to discovery of new anticancer-drug lead compounds. It has been facilitated by the creation of small molecular profiles and chemical-structure databases and a complete annotation of chemical-genetic profiles based on well-genomic characterized cell lines. Weinstein et al. (1997) at the U.S. National Cancer Institute (NCI) developed the first high-throughput assay-based on 60 cancer cell lines, named NCI-60, for screening cancer candidate drugs. The access to the NCI-60 database is via the CellMiner web-based application^3^ (Reinhold et al., 2012, 2015). Various other researchers’ groups developed similar pharmacologic and biochemical approaches to screening compounds in large number of cell lines derived from various types of cancers. The results of these large pharmacogenomics studies are compiled in the following platforms: the Cancer Cell Line Encyclopedia (CCLE^4^; Barretina et al., 2012), Connectivity Map (CMAP^5^; Lamb et al., 2006), Genomics of Drug Sensitivity in Cancer (GDSC^6^; Garnett et al., 2012) and the Cancer Target Discovery and Development Project^7^ Advances in high-throughput technologies have allowed improvements and expansions in these data sets as described in details elsewhere (Dan et al., 2002; Basu et al., 2013; Yang et al., 2013; Covell, 2015; Klijn et al., 2015; Iorio et al., 2016). Together, these website platforms display genomic and pharmacogenomics datasets of over 1,000 cancers cell lines and their responses to more than 25,000 therapeutic agents.

This review will consist of an introduction of pharmaco genomics datasets for exploring potential inter-relationships among cancer genomics and drug discovery for precision cancer medicine. First, we will introduce cBio Cancer Genomics Portal (cBioPortal) and discuss the key molecular and clinical features using some examples of cancer genomics datasets. Second, we will exemplify how to query cancer candidate genes and small molecules and chemical with advanced web bioinformatics profiling tools available in CellMiner and Connectivity Map platforms. We illustrate in each section examples of relevant studies showing potential inter- relationships among cancer genomics and drug discovery. Finally, we discuss on challenges, limitations and new directions to personalized gene-targeted cancer therapy.

## cBioPortal

cBioPortal^8^ provides bioinformatics tools for gene-based visualization and analysis of molecular profiles and clinical attributes of cancer patients obtained in the large-scale clinical studies (Cerami et al., 2012; Gao et al., 2013; Schroeder et al., 2013). The portal was developed at Memorial Sloan-Kettering Cancer Center (MSKCC) Computational Biology Center (cBio) in partnership with The Cancer Genome Atlas (TCGA) and the International Cancer Genomics Consortium (ICGC). Currently users can access data from more than 10,000 tumor samples of 126 studies (as July 2016) and many provisional cancer studies are continuously updated. The data set includes DNA copy number variation, DNA methylation values, mRNA and microRNA expression based on microarrays, mutation profiles, protein and phosphoprotein levels when available for that study. The clinical data available include overall survival and disease-free survival intervals, gender, age, stage, and tumor grade. Recently, the portal has added digital images of each patient tumor biopsy displaying their histological and proliferative patterns. The cBioPortal provides Pathway Commons tools for exploring biological networks and interactions with up to 50 of the most highly altered neighboring genes in the selected cancer study. Biological networks can be viewed using cytoscape^9^ tolls for visualizing molecular interaction networks and integrating these interactions with gene expression profiles of interest. Furthermore, using the Genes and Drugs menu, users can predict drug-target interactions in a network.

The cBioPortal platform presents a list of over 70 scientific articles reporting on the growing number of clinical studies available in portal database for interactive data analyses (Cerami et al., 2012; Gao et al., 2013). CBioPortal has been cited in hundreds studies to describing mutational patterns of most common oncogene and tumor suppressor, insertion, deletion, and amplification that drive specific tumor-type, and prognosis in patients and entire cohort (Van Allen et al., 2013; Lawrence et al., 2014; Forbes et al., 2015).

### Short Protocol for the Use of the cBioPortal

**FIGURE 1:**
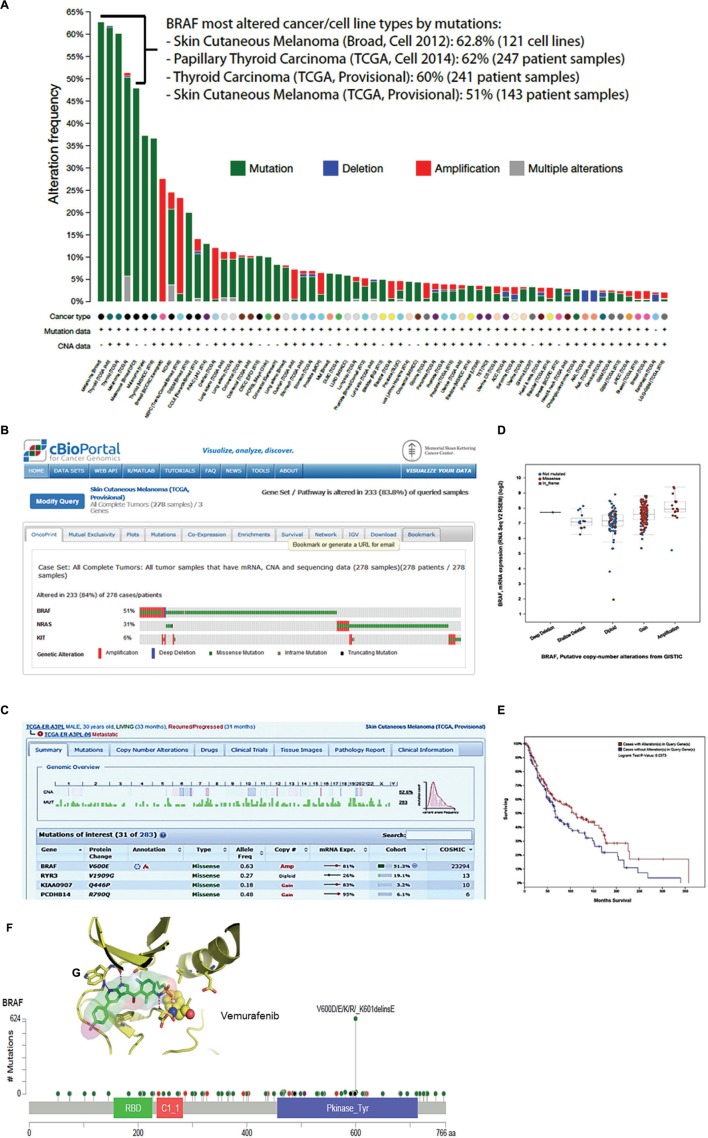
**cBioPortal data visualization and analysis.** **(A)** Genomic alteration frequency in the BRAF gene in 123 cancer patient studies, NCI-60 and CCLE-883 cancer cell lines. **(B)** Oncoprint showing frequencies of genomic alterations observed in BRAF, N-RAS, and c-KIT gene in 278 tumor samples in the TCGA skin cutaneous melanoma. **(B)** The panel shows that 143 (51%) of patients of the skin cutaneous melanoma TCGA study had one or more BRAF alterations. **(C)** The panel shows a genomic overview of one patient identified by the number TCGA-ER-A3PL-06. The upper view panel shows copy number variation and the frequency of mutations observed in each chromosome of the patient. The lower view panel shows details of the four top genes from 283 genes in which at least one alteration was identified. **(D)** The plot shows the correlation between BRAF mRNA expression and the putative copy-number alterations (gain or amplification). In this plot is include the deep or shallow deletion (not mutated, missense and in frame mutation) BRAF mutation. **(E)** The Kaplan–Meier overall survival curve indicates that the cases with BRAF mutation had higher overall survival than the cases without BRAF alterations. **(F)** This schematic representation shows the position and hot spot mutations across BRAF protein domains. The missense mutations most frequently observed are V600E, D, K, and R. Inside **(G)** shows a stick model representation for BRAF kinase domain and molecular inhibitory mechanism proposed to vemurafenib (Zelboraf; Plexxikon/Roche), a small molecule drug approved to treat melanoma patients.

A stepwise protocol containing the instruction and guidance for querying the database is described in tutorial of the cBioPortal website. The user needs to follow up four-steps in the cBioPortal web interface and click on specific bottom to select: (1) a cancer study of interest, for example, skin cutaneous melanomas studies; (2) one or more genomic profiles, for example, mutations and copy number alterations; (3) a patient case set, for example, all complete TCGA patients with mutation, copy number, and mRNA data; and (4) a gene of interest using HUGO gene symbols or gene sets or pathways of interest. In the example in **Figure 1**, we entered BRAF gene and selected “All Cancer Studies.” Based on the user input, the portal automatically generates a complete Oncoprint, which is concise and compact graphical summary of genomic alterations. In this report there are boxes to access data sets, which include mutations, mRNA expression, DNA methylation, clinical outcome, biologic pathways, network neighborhood, and several other parameters. In addition, the users can inquire on the expression of special set genes associated with cell cycle control (34 genes), DNA damage response (DDR; 12 genes), and other genes involved in the canonical signaling and pathways of the cells. Most of data generated can be exported as PDF or SVG file.

**Figure 1A** shows the result of one query to interrogate overall genomic alterations frequency in BRAF gene in all 123 oncogenomic datasets. The columns display in color the frequency of each alteration (mutation, deletion, amplification, and multiple) observed in 63 out of 123 studies deposited in cBioPortal. We highlighted four studies performed by TCGA and Broad Institute of MIT and Harvard Medical School, in which BRAF may be the key driver gene of skin cutaneous melanoma and thyroid carcinomas. In these studies, BRAF genomic alteration frequency ranged from 51 to 62.8%. In panel B, we show the oncoprint report for retrieval in which we inquired for co-occurrence of genomic alterations in BRAF, N-RAS and c-KIT genes in 278 total cases of skin cutaneous melanoma analyzed by TCGA provisional study (not published). The results show a statistical significance for mutual exclusivity between BRAF and N-RAS within the patient cohort. The same was not observed for N-RAS and c-KIT. BRAF missense mutation occurred in 51% of population. Interesting, one third of patients displayed both amplification and mutation in BRAF gene (columns in red and green). The genomic overview of one patient from this study identified by the number TCGA-ER-A3PL-06 is presented in **Figure 1C**. The upper view of this panel shows copy number variation and frequency of mutations observed in each chromosome of this particular patient. The lower view panel displays details of the four top genes from 283 genes in which at least one alteration was identified. A plot shows the correlation between BRAF mRNA expression and the putative copy-number alterations (gain or amplification; **Figure 1D**). The Kaplan–Meier overall survival curve (**Figure 1E**) indicates that the cases with BRAF mutation had higher overall survival than the cases without BRAF alterations (Logrank Test *p* = 0.0373). **Figure 1F** shows hot spot mutations across BRAF protein amino sequence and highlight frequently observed missense V600D/E/K/R mutations. **Figure 1G** shows a stick model representation of BRAF kinase domain and its inhibitor Vemurafenib bound to ATP-binding site of the active kinase. The cBioPortal tools also allow the users to identify cancer mutational landscapes using the Mutation Assessor application, predicting if a genetic alteration may impact to clinical outcomes.

### Advantages and Limitations

By integrating multiple cancer genomics projects, cBioPortal enables the users to analyze complex data sets and translate into biologic insights and immediate clinical applications. The Cancer Genomic Data Server (CGDS) and Web API interface allow the use of many programming language, such as Python, Java, Perl, R or MatLab, as well as the Integrative Genomics Viewer, which is an external link. All these applications and statistical tools allow the development of predictive models and meaningful interpretation of molecular and clinical data. The cBioPortal has also many options for saving, downloading, and sharing results from a query. Since most of the studies in cBioPortal have been published, the users can download the original articles for further interpretation across relevant datasets.

Next-generation sequence (NGS) technologies for whole-genome and exome sequencing continue to increase in quality and accuracy to capture complex genomic alterations in DNA molecules. Nonetheless, various cancer genome data were obtained using different protocols from different laboratories and many technical and statistical issues remain unsolved. DNA is isolated from diversified heterogeneous tumor and normal tissues with create potential bias to estimate the relative proportion of the germline mutation, *de novo* variants and rare mutated alleles in a sample. To overcome this problem, it will be necessary to analyze separately whole exome sequencing of tumor tissue-associated fibroblast cell lines, circulating tumor cells (CTCs) in human blood extracts and single cell clones from tumor tissue to enhance accuracy of oncogenomic data. These approaches should become a priority in future studies.

### CellMiner

CellMiner^10^ is a web-based suite of bioinformatics tools designed to explore the drug activity in the NCI-60 cell lines (Reinhold et al., 2015). The database is continually updated and maintained by Center for Molecular Therapeutics (CMT) and Developmental Therapeutics Program (DTP) of The U.S. National Cancer Institute (NCI) (Weinstein et al., 1997; Reinhold et al., 2012, 2015). The NCI-60 cell lines have been characterized previously regarding tissue of origin, age and sex of patient, histology, DNA ploidy, p53 status, multidrug resistance function, and doubling time. The results of the comparative genomic hybridization and karyotypic analysis, DNA fingerprinting and mutation analysis as well as the levels of RNA and miRNA transcript expression and protein and phosphoprotein, amino acid changing genetic variants, protein function affecting genetic variants in each cell line have been described in previous publications (Nishizuka et al., 2003; Shankavaram et al., 2007; Reinhold et al., 2012, 2015; Abaan et al., 2013; Reinhold et al., 2014; Varma et al., 2014). CellMiner tools allow rapid data retrieval of transcripts for 22,379 genes, 92 proteins and 360 microRNAs along with activity reports for more than 20,503 chemical compounds, which include 102 drugs approved by the U.S. Food and Drug Administration (FDA). In addition, quantitative proteome and kinome profiles of the NCI-60 panel covering over 10,350 proteins and 375 protein kinases are available^11^ This supplementary website displays protein and peptide expression of 59 cell lines of NCI-60 panel allowing comparison of differentially expressed proteins between samples (Gholami et al., 2013). The database serves as reference to query the abundance and distribution of proteins in each cell line as well as protein signature for drug sensitivity and resistance (Gholami et al., 2013; Reinhold et al., 2015).

The rcellminer is an R package that provides a wide range of functionality to help R users to access and explore molecular profiling and drug response data in the NCI-60 CellMiner platform (Luna et al., 2016). This tool allows many statistics and visualization analysis such as clustering sets of drugs with similar mechanisms in heat maps to show their inter-relatedness, and calculate correlations between gene expression, mutations or deletion, and drug activity profiles. The *Z*-score is the number of standard deviations away from the mean of expression and the average *z*-score for each cell line is presented in the histograms. For instance, the use of *z*-score average has confirmed that broad chemotherapeutic resistance among NCI-60 cell lines correlates with expression of MDR1 (Alvarez et al., 1995). Many other studies using different types of cell lines have confirmed literature results linking some specific gene mutations to EGFR, ERBB2, MET, PDGFR, ALK, and BRAF gene with the NCI-60 cell line sensitivity to kinase inhibitors (Paull et al., 1989; Ikediobi et al., 2006; Shoemaker, 2006; Quintieri et al., 2007; McDermott et al., 2008).

Due to the broader role of the DNA damage response (DDR) in cancer cell response to cytotoxic agents (Jeggo et al., 2016), a recent study has examined the relationship of 260 DNA repair genes with overall drug responses in the NCI-60 cell lines (Sousa et al., 2015). The authors identified, as expected, that the checkpoint genes TP53, ATM (ATM serine/threonine kinase), ATR (ATR serine/threonine kinase), MLH1 (mutL homolog 1), MSH3 (mutL homolog 3), and APC (adenomatous polyposis coli) were frequently mutated in these cell lines (Sousa et al., 2015). Cell lines with homozygous deleterious mutation, named as putative genetic knockout cell lines, were selected and used as control to validate the predictive values of DNA repair genes to DNA damaging drug activity (Sousa et al., 2015). The results indicated a significant association between the Fanconi anemia genes (FANCM, FANCP/SLX4, FANCI) and the activity of alkylating agents, antifolates, topoisomerase II and DNA synthesis inhibitors. Other significant associations between specific DNA repair genes and drug activity were RAD51C (FANCO) and UBE2N whose expression had a positive association with topoisomerases I and II and DNA synthesis inhibitors. In addition, this study confirmed the pivotal role of Schlafen family gene SLFN 11 as major predictor of 147 clinically relevant DNA damaging agents including topoisomerases I and II inhibitors, alkylating agents and DNA synthesis inhibitors (Zoppoli et al., 2012; Sousa et al., 2015). Functional studies for synthetic lethality in yeast have identified mutually dependent partners or pair of DNA repair genes that work together to transduce the DNA damage signals and survival of normal cells (Chan and Giaccia, 2011; Nijman, 2011). CellMiner database is a useful tool to infer loss-of-function through heterozygous or homozygous mutation and loss of expression during the screening of DNA damaging agents across NCI60 cell lines. Potential pairs of human genes present in certain cancer cell lines can help the development of new drugs for synthetic lethality chemical approach.

### Short Protocol for the Use of the CellMiner Database

**FIGURE 2:**
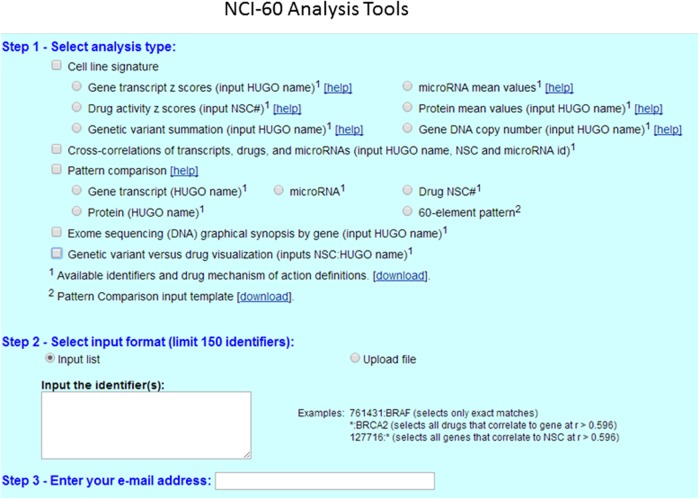
**CellMiner interface for querying genomic data and drug activity in the NCI-60 database.** CellMiner web interface is accessible by clicking on the NCI-60 Analysis Tools tab. The users select in step1 the square box for cell line signature, and next clicking to one of the radio boxes, including gene transcript *Z*-scores, drug activity Z-scores, or genetic variant summation. The users can check the square box for pattern comparison analysis, and choose one of the radio boxes for gene transcript, protein or Drug NSC number. The specific identifier or pattern of interest is selected in step 2, either by typing directly one gene symbol or one NSC# drug number from the list. Alternatively, the query can be done using the “Input list” function, or by uploading a file using the “Upload file” function. A maximum of 150 identifiers (genes, microRNAs, or drugs) can be input at once. The result is instantly e-mailed to the address entered in step 3.

**FIGURE 3:**
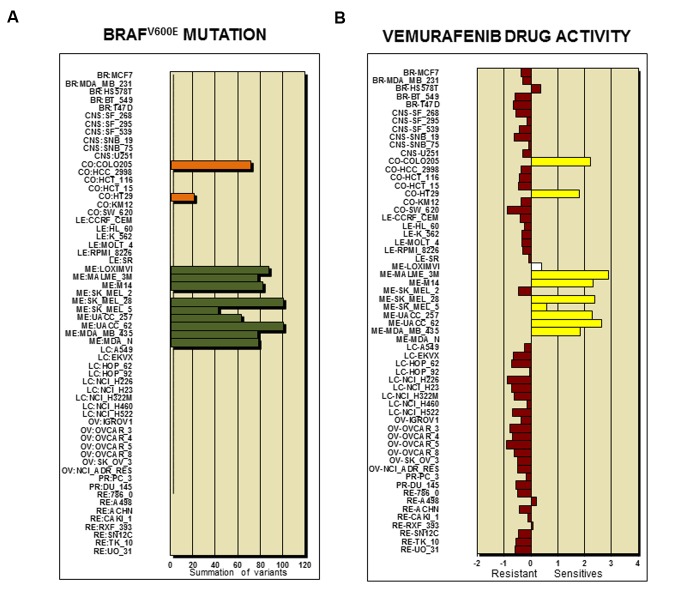
**BRAF genetic variant and sensitive to drug activity.** **(A)** Frequency of BRAF^V 600E^ mutation across the NCI-60 cell lines. The *y*-axis shows name of cell line and *x*-axis shows “Summation of Variants.” Two colon cancer cell lines (CO:COLO205 and CO:HT29) and nine melanoma cell lines (ME:LOXIMVI, MR:MALME_3M, ME:M14, ME:MEL_28, ME:SK_MEL5, ME:UACC_257, ME:UACC_62, ME:MDA_MB_435 e ME:MDA_N) are positive for BRAF^V 600E^ mutation. **(B)** Vemurafenib drug activity in the NCI-60 cell lines. The bar graphic shows the *Z*-score for sensitive (0 to +3) and resistant cell lines (0 to -3). The results indicate that 8 out of 9 melanoma cell lines and 2 out 7 colon cancer cell lines responded to the treatment according to BRAF^V 600E^ mutation status depicted in **(A)**.

**FIGURE 4:**
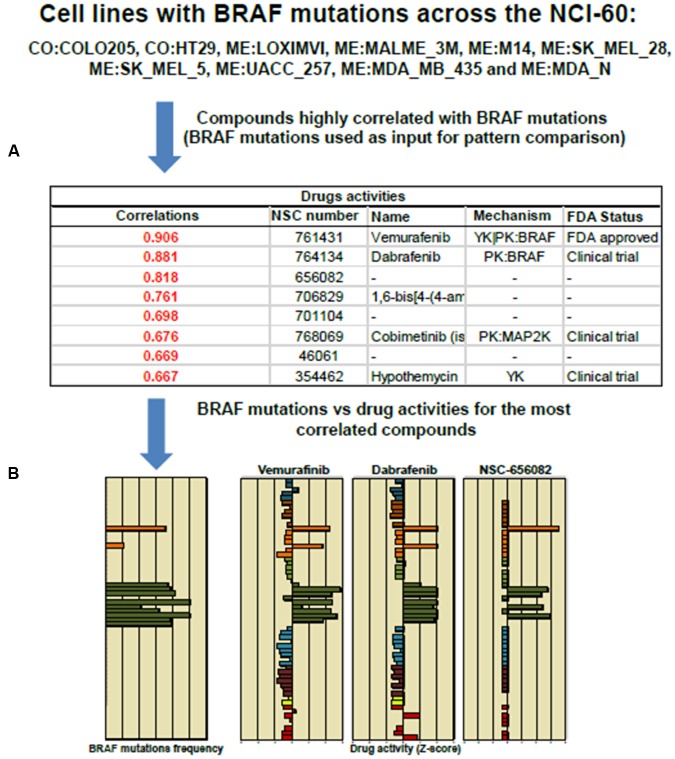
**Pattern comparison in the NCI-60 cell lines to significant drug correlation using as an input Vemurafenib and BRAF mutation.** **(A)** The table displays NSC, names and FDA status for the top eight compounds highly correlated to Vemurafenib activity. **(B)** The bar graphs shows the “*Z*-score determination” for the top three compounds: Vemurafenib, Dabrafenib and NSC 656082, respectively. Most of cell lines harboring BRAF^V 600E^ mutation responded positively to Vemurafenib and Dabrafenib. The cell line responses were distinctly different to NSC 656082 compound.

**Figure 2** shows the analysis tools tab with currently accessible analytical parameters of the CellMiner platform. A step by step to set up a protocol for querying and retrieval information in NCI-60 database is described in great detail elsewhere (Reinhold et al., 2015). The platform is continually updated in new parameters and tools have been implemented recently (Reinhold et al., 2014, 2015). To illustrate CellMiner applicability we show in **Figure 3** results of correlations of mutated BRAF gene expression and Vemurafenib drug activity against NCI-60 cell lines (**Figure 3**). There are in the list colon cancer cell lines: CO:COLO205 and CO:HT29 and melanoma cell lines: ME:LOXIMVI, MR:MALME_3M, ME:M14, ME:MEL_28, ME:SK_MEL5, ME:UACC_257, ME:UACC_62, ME:MDA_MB_435 e ME:MDA_N. Next, we used the toll “pattern comparison” to discovery similar drugs to target mutated BRAF (**Figure 4**). To submit the first job (**Figure 3A**), we checked two boxes in step 1, at first “cell line signature” (square box), and next “genetic variant summation” (radio button). In step 2, we entered the gene symbol; in this case, “BRAF.” To submit the second job (**Figure 3B**), in step 1, we checked the box “genetic variant vs. drug visualization.” Then, in step two, we entered the NSC number: gene symbol, in this case, 761431:BRAF. NSC number of Vemurafenib is 761431. This number can be found in the list of identifiers and drug mechanism that is available for download (step 1 CellMiner interface tools).

The tool “Pattern Comparison” allows comparisons to identify compounds with similar toxic activity against NCI-60 cell lines based on “*Z*-score determination.” To submit a job, in the step 1, we checked first the box “Pattern Comparison,” next, a radio button for Drug NSC. In the step 2, we typed Vemurafenib NSC number 761431. The CellMiner retrieval show an Excel file containing a list of 725 compounds. The table in **Figure 4** shows the top 8 NSC numbers for the highly correlated compounds, including the names if available, and its mechanism and FDA status. The drug Dabrafenib/GSK2118436 with a correlation of 0.881 is in fact a BRAF-targeted drug approved in clinical trial to treat melanoma patients. The bar graphic showing *Z*-score determination for each compound as compared to Vemurafenib is shown in **Figure 4**. It is important to mention that Vemurafenib and Dabrafenib have achieved clinical approval against BRAF^V 600E^ melanoma patients. However, colon cancer patients with BRAF^V 600E^ do not respond to these drugs (Solit and Rosen, 2014). The list of cell lines that responded to BRAF inhibitors is consistent with GDSC database (Yang et al., 2013). The query is described as in **Figure 3B**. In step 1, we checked the box “genetic variant vs. drug visualization.” Then, in step two, we entered NSC number:gene symbol, in this case, 764134:BRAF. NSC number of Dabrafenib is 761431.

### Advantages and Limitations

Cell miner is a powerful and friendly tool to perform chemical-genetic profiling based on gene and drug activity. The exploration of CellMiner database has allowed the discovery of mechanisms of action of uncharacterized and structurally similar compounds (Shankavaram et al., 2007; Reinhold et al., 2014, 2015). The use of *z*-score as normalization index facilitates integration of data such as gene transcript expression levels and drug activity rates. The Pattern Comparison algorithm is another advanced tool developed by the NCI-60 group that enables investigators to search for compounds or molecular targets with similar patterns of activity in the NCI-60 cell lines (Paull et al., 1989; Zoppoli et al., 2012; Reinhold et al., 2015). By comparing their chemical-genetic profiles and hierarchical cluster analysis according to mechanistic category of anticancer agents, potentially important associations can be identified between cancer-specific genomic alterations and pharmacological responses (Holbeck et al., 2010). Moreover, users can identify if the presence wild-type or mutated p53 gene expression influences drug cell death activity. A new tool named “genetic variation and drug visualization” allows the users to query on new compounds that display similar specificity to mutated proteins (Reinhold et al., 2015). We exemplified the use of this tool using mutated BRAF and Vemurafenib to discover that Dabrafenib has same molecular target (**Figure 4**).

One limitation in the NCI-60 database is that chemotherapeutic compounds were tested at a single dose and cytotoxic or cytostatic effects were determined at 48 h without considering cell doubling time and cell cycle stage of a cell line. The screening was done in a small number of cell lines representing the nine tissue-derived cancer cell lines and therefore few tumor subtypes. There are a number of compounds that induces cell death via chemical reactivity and membrane disruption and should be clustered separately to indicate a non-genetic mechanism. Some other discrepancies in the multiple comparative pharmacogenomics studies were discussed elsewhere (Haibe-Kains et al., 2013; Weinstein and Lorenzi, 2013).

## Connectivity Map

The Connectivity Map^12^ uses the concept of connectivity based on compound-gene signature developed by Todd Golub’s group at Broad Institute of MIT, Whitehead Institute and Harvard Medical School, Massachusetts (Lamb et al., 2006; Lamb, 2007). In their first study, the group examined the effects of exposure cancer cell line MCF-7, HL-60, SKMEL5 and PC3 to 164 perturbagens on gene expression using Affymetrix GeneChip microarrays. Perturbagens are a small molecule or a genetic interference such as knockdown or overexpression of a gene, using a genetic reagent such as shRNA (short hairpin RNA) and CRISPR/cas9 system. The instance is the basic unit of data obtained in one treatment, the source, the concentration, the cell line used, and the scan numbers for the treatment and its control (Lamb, 2007). For comparing two samples and to determine cumulative probability function by the null hypothesis, CMAP uses Kolmogorov–Smirnov test. CMAP database contains the results obtained with 1309 compounds and more than 7,000 core reference expression profiles. To date, the Broad Institute’s LINCS program has expanded the compound collection to over 20,000 using 50 types of cells, which gave over 1,800,000 perturbation profiles^13^ Differentially expressed genes between disease and normal conditions were used to form a signature for the disease. Some of these experiments were done using a hybrid capture sequencing method, which examine mRNA expression levels of 1,000 landmarker genes.

These genes are minimally redundant and widely expressed in different cellular contexts. The CMAP 2.0 software identifies chemicals with similarity in gene profiling among the matched genes or query gene signatures that were previously identified as common gene-expression changes to one or more known compounds of the CMAP database. The software reveals both compounds with positive and negative connectivity using the up-regulated query genes and down-regulated query genes representing a biological process. As a result of the comparisons, all drug profiles in the reference database will be given a connectivity score range from 1 to -1 representing their connections to the query signature. The method also allows the exploration of gene signatures of cellular states, development, and disease. Thus, CMAP can predict the regulatory networks and molecular interactions that take place in different types of cells under various conditions (Qu and Rajpal, 2012).

Yu et al. (2015) use CMAP to find key apoptosis genes induced after exposure to 191 anticancer drugs. The authors identified BCL2L11 (also named as BIM) and TNFAIP3 (also named zinc finger protein A20) among 13 top critical regulators of anticancer agents induced cell death. Cancer cells resistant to many cytotoxic compounds have been linked to widespread occurrence of deleted or down-regulated gene of the extrinsic signaling pathways, such as TNF family members and up-regulation or amplification of BCL2 protein family members of the intrinsic mitochondria pathways. In fact, a recent study using CMAP showed that LY294002, a PI3K inhibitor, and gossypol and AT-101, MCL1 or other anti-apoptotic BCL-2 family member inhibitors, were capable of reversing the prednisolone-resistance of MLL-rearranged Acute Lymphoblastic Leukemia (ALL) in infants (Spijkers-Hagelstein et al., 2014). Finally, CMAP database has been used to drug repositioning which is a rational screening to identify and compare drug efficacy and side effects of existing drugs to specific diseases in concert with gene signature (Hu and Agarwal, 2009; Qu and Rajpal, 2012).

### Short Protocol for the Use of Connectivity Map

A stepwise protocol for querying the database is described in Connectivity Map webpage^14^ after a user has logged in. For querying, the user needs to convert the list of genes to be query into correspondent probeset of the Affymetrix array. This can be done using tools available at http://www.affymetrix.com/analysis/netaffx/index.affx. There is a “help page” explaining how to make a .grp file from a tag list. The list of .grp files (add to Microsoft Excel file) of up- and down regulated genes is uploaded separately in the query. The permuted result page shows a total of instances and functionally or structurally similar perturbagens best connected (positively and negatively) along with it the connectivity score, which is a combination of the up score and down score. High (positive or negative) connectivity score correlates with low p-score (permutation score).

**FIGURE 5:**
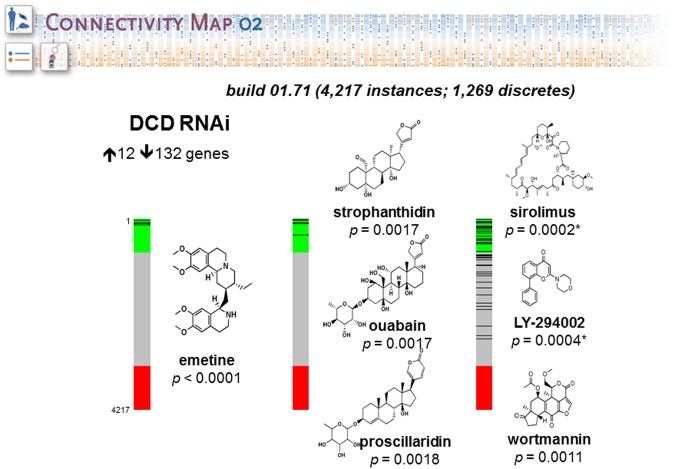
**Connectivity Map portal retrieval.** The figure shows barview icons of a query to interrogate potential relationship between the mRNA expression profiles induced by Dermcidin (DCD) and those induced by drugs and small molecules of CMAP database. The barview icons were constructed using 4,217 instances, each representing an individual treatment instance. The results were ordered by their corresponding connectivity scores and *p* levels. The data support the prediction that DCD-induced gene signature is strongly correlated with signatures induced with small molecules LY-294002, wortmannin and sirolimus, which are potent inhibitors of PI3K, AKT and mTOR signaling pathways, respectively. The chemical structures were obtained from ChemBank. The figure was reproduced with the permission from Moreira et al. (2008).

We have done a study to evaluate the role of Dermcidin (DCD) in breast cancer tumorigenesis using as model MDA-MB-361, a breast carcinoma cell line widely used to investigate breast cancer pathobiology (Moreira et al., 2008; Bancovic et al., 2015). This work was aimed at finding the connections between a molecular signature induced by DCD in breast cancer and drugs that are likely to share some common molecular mechanism. We collected and analyzed the global mRNA expression levels of MDA-MB-361-pLKO (control) and MDA-MB-IBC-I expressing shRNA against DCD (treatment) using Affymetrix U133 Plus 2.0 chip. Next, we compared 132 up-regulated and 12 down-regulated genes in the dataset generated using dCHIP software. We uploaded separately the list of genes up and down-regulated. CMAP identified connectivity with a similar set of genes (signature) generally elicited in cellular response to the drugs Sirolimus (*p* = 0.0002), LY-294002 (*p* = 0.0004), and Wortmannin (*p* = 0.0011) (**Figure 5**). These compounds are specific inhibitors of mTOR (Mechanistic target of rapamycin), PI3K (Phosphatidylinositol-4,5-bisphosphate 3-kinase) and AKT (V-Akt murine lymphoma viral oncogene homolog), respectively. This result suggests that DCD controls the expression of same set of genes modulated by these inhibitors and may exert related effects on the cells.

### Advantages and Limitations

CMAP offers simple Web interface tools that allow users to query gene signatures from a large number of small molecule compounds. The comparative studies using parenteral and gene knockout cell lines and their expression profiles have shown that small molecules clustered with knockouts of targeted genes (Lamb et al., 2006). The CMAP collection incorporated thousand drugs able of inducing or reversing diseases states based on genes up- and down regulated. This dataset has allowed the discovery of unknown off-targets or unknown disease mechanisms of great clinical interest. The portal can automatically generate cross queries and identify structure-effect relationships based on chemical-induced gene expression profiles which translate molecule’s capacity to modulate the function of protein-network components. The biological interpretation of predicted results can be confirmed using the gene ontology, associations and relationships of genes with diseases and biological pathways on whole organisms provided by various resources like the Omin database^15^

One disadvantage is that compounds-signature profiles are based on measurements of 1,000 landmarker genes in few human tumor cell lines derived from breast, prostate, and leukemia. Various experimental bias including off-target transcriptional effects, specific genomic alterations of cancer cell types, doses, and time points may impact on cell-line-specific response to drugs. Additional molecular features to improve the power of CMAP, such as comparison in the levels and modification (phosphorylation) of proteins of activated signaling pathways are necessary to confirm correlative or mechanistic approaches being observed for all targeted agents and experimental conditions.

## Concluding Remarks and New Directions

Over the past 15 years, technological and bioinformatics advances have made possible the integration of multiple omics datasets and application of meta-analysis bioinformatics and systems biology approaches to analyze complex biological networks of cancer genomic datasets. As results, hundreds of novel oncogenes, tumor suppressor genes, DNA repair genes, focal adhesion, integrin signaling, extra-cellular matrix, actin/cytoskeleton genes have been retrieved from these cancer genome databases (Cline et al., 2013; The Cancer Genome Atlas Research et al., 2013; Lawrence et al., 2014; Forbes et al., 2015). The molecular, clinical and epidemiological features of over 126 large scale cancer types have been studied (as July 2016) and they are available for research community in cBioPortal platform. The well-known oncogenes and tumor suppressors TP53, EGFR, PIK3CA, PTEN, APC, KRAS, and BRAF have broadly used to classify tumor pathologies and tumor subtypes (Lawrence et al., 2014). This has been the basis for development of global new patient-centric clinical targeted therapy and precision oncology in development nowadays (Biankin et al., 2015). A large number of molecular aberrations and multiple recurrent chromosomal gains and losses identified in these oncogenomic databases remain unknown and represent important open question for investigation (Cline et al., 2013; Lawrence et al., 2014).

The tumor’s molecular signature varies widely in several human malignancies and spectrum of gene mutations that may have potential as predictive biomarkers continues to grow (Chang et al., 2016). From a clinical point of view, a major challenge in the interpretation of mutation profiles is to assess genomic variants that impact on drug treatment and clonal evolution. Recently, COSMIC Web portal updates on 2 million mutated variants in over 1 million tumor samples in the cancer genome examined (Forbes et al., 2015). We know that a very small number of rare mutations substantially contribute to oncogenesis and cancer progression. How many mutations could be made druggable? We do not know. What we know is that pathway-targeted therapies for many diverse variants converge on similar deregulated sub-pathways. For instance, cutaneous skin melanoma cells bearing BRAF^V 600E^ mutation that respond positively to Vemurafenib, the first BRAF kinase inhibitor approved to treat metastatic melanoma, progress to resistant cells (Bollag et al., 2012; Sullivan and Flaherty, 2013). The resistant cells display activation of parallel signaling pathways, such as ERK signaling, through KRAS and BRAF^V 600E^ amplification and mutations in the MAP2K1 and MAP2K2 genes (Solit and Rosen, 2014). Clonogenic growth of resistance BRAF-mutant melanoma cells treated with a combinatory drug therapy using BRAF and MEK inhibitors activate parallel P13K/AKT pathway (McCubrey et al., 2012; Solit and Rosen, 2014). There are many other similarities in clonogenic resistance mechanisms induced by receptor tyrosine kinase inhibitors in different types of tumors and cell lines (McCubrey et al., 2012; Wilson et al., 2012; Obenauf et al., 2015; Campbell et al., 2016; Kirouac et al., 2016). It is interesting that not only genetic mutation and amplification of these driver oncogenes, but also changes in cellular environment increase dependency in Ras/Raf/MEK/ERK and PI3K/PTEN/Akt/mTOR cascades (Campbell et al., 2016; Kirouac et al., 2016). How many sub-pathways are there? It is not yet imaginable. These results drive up some concerns on the promises of predictive genetics of drug sensitivity in the current personalized cancer medicine (Rubio-Perez et al., 2015).

The CellMiner and Connectivity Map are two examples of the multi-dimensionality cancer genomic platforms for predicting and developing potential targeted strategies to cancer cells. Using a variety of well-established cancer cell lines and *in vitro* and *in vivo* experiments and systems pharmacology analysis, several molecular mechanisms, pathways and cellular processes directly affecting drug response to a larger number of anticancer compounds have been revealed (Yang et al., 2010; Sharma et al., 2010; Heiser et al., 2012; Covell, 2015; Goodspeed et al., 2015; Reinhold et al., 2015). To further evaluate the suitability of any particular cell line as a model, CCLE and CDSC consortiums’ groups have undertaken deeply Integrative genomic and transcriptomic analyses of more than 1000 cell lines (Barretina et al., 2012; Klijn et al., 2015; Stransky et al., 2015; Iorio et al., 2016). From their reports it was clear that most cell lines display stable genomes and conserve similarly to molecular subtypes seen in patient’s tumor (Barretina et al., 2012; Klijn et al., 2015; Stransky et al., 2015; Iorio et al., 2016). Nonetheless, studies of RNA-Seq gene expression profiles demonstrated the presence of over 2,200 gene fusions in 232 cell lines. Among them, 168 are well known canonical oncogenic fusions, and more than 1,400 are new fusions involving a wide variety of gene patterns. In addition, it was observed that many human cell lines have acquired DNA and RNA fragments from human and murine viruses. More important, these studies confirmed a co-expression of transcripts to MET, EGFR, ITGA3 (Integrin α3), EPHA2 (the ephrin-A receptor 2), and CAV2 (Caveolin 2) gene among many cell lines (Klijn et al., 2015). This suggests that these cell lines have a constant activation of PI3K/AKT or MAPK/ERB signaling pathways, as described in many studies (Campbell et al., 2016; Kirouac et al., 2016). These popular cancer cell line models are of fundamental importance and reliable tools for implantation of systems pharmacology approaches (Keith et al., 2005; Yang et al., 2010). Nonetheless, predictive models for the complex combination of genetic alterations and biochemical pathways that translate in drug sensitivity and resistance in real solid tumors remain a challenge (Keith et al., 2005; Yang et al., 2010; Domcke et al., 2013; Covell, 2015).

In order to make tumor cells sensitive to drugs, we need to understand the role of programmed cell death proteins and mechanisms they use for re-wiring signaling pathways that control metabolism, cell growth, proliferation, invasion and cell-to-cell variability (Yang et al., 2010; Xia et al., 2014). Drug sensitivity varies according to the levels of concentration of paracrine and autocrine ligands, nutrients and metabolites present in the tumor microenvironment (Yang et al., 2010; Xia et al., 2014; Obenauf et al., 2015). The tumor microenvironment consists of mix population of cancer cells, stromal cells, vascular cells, and inflammatory cells growing under an acidic and low oxygen metabolic condition (Dang, 2012). Few *de facto* chemotherapeutic agents exist for modulating tumor metabolism (Galluzzi et al., 2013). Much remains to be learned about cancer metabolic rewiring and the impact of oncogenes and tumor suppressor genes, as examples c-Myc, Ras and p53, in the pentose phosphate pathway, redox homeostasis and mitochondrial respiration in cancer cells, and non-malignant cells in fully tumor conditions *in vivo* (Galluzzi et al., 2013). Translating these *in vitro* results to *in vivo* is not easy because of biochemical and genetic differences between cultured cell lines and heterogeneity within tumor cell populations.

Novel *in vitro* and *in vivo* approaches have been developed that reproduce heterogeneous behavior and genetic diversity of tumor cells that determine the evolution of resistance clones in response to chemotherapy (Garnett et al., 2012; Goodspeed et al., 2015). Conditional reprogramming (CR) induced by a Rho kinase inhibitor (Y-27632) is an emerging cell culture technology for generation of patient-derived stable cell lines and explore their genetic diversity in organoid 2D and 3D cell culture assays (Liu et al., 2012). Patient-derived tumor xenografts are potential complementary models for screening and development of anti-cancer agents *in vivo* (Williams et al., 2013; Hidalgo et al., 2014). The proof of concept that it is possible to successfully apply PDXs as model has been provided in many large-scale pharmacogenomics PDX studies using various cancer types (Dey et al., 2013; Hidalgo et al., 2014). The development of breast carcinoma subtypes-in-mouse PDX models has helped the identification of small chemical inhibitors to PI3K (Lehmann et al., 2014), checkpoint kinase 1 (Ma et al., 2012), aurora kinase (Romanelli et al., 2012), BCL-2 family-BH3 mimetic (Whittle et al., 2015), and many other drugs approved for treatment of breast cancers (Zardavas et al., 2013). In a recent study, Krepler et al. (2015) generated 12 PDXs of BRAF resistant melanoma patients. The authors applied genomic and proteomic methods to reveal the major signaling pathways and possible drivers of resistance in the PDXs. For instance, NRAS mutations were found in 3 PDXs, MAP2K1 (MEK1) mutations in 2, BRAF amplification in 4, and aberrant PTEN in 7 (Krepler et al., 2015). Furthermore, the authors analyzed potential combination of BRAF/MEK inhibitors Encorafenib and Binimetinib, or a triple combination of both inhibitors plus pan-PI3K inhibitor BKM120. They observed that only triple combination exceptionally abrogated tumor growth in PDX models (Krepler et al., 2015). Thus, this co-clinical model can be used to refine precision medicine approaches and identification of biomarkers for patient clinical outcome.

The CSCs, or cancer initiating cells, has emerged as target of new therapeutic modality aiming at overcome drug resistance in clinical cancer treatment (Frank et al., 2010; Egeblad, 2011; McGranahan and Swanton, 2015; Xie and Bourne, 2015). CSCs are characterized by their increased drug efflux capacity, which is mediated by ATP-driven ABC drug transporters (Dean et al., 2005). Verapamil and Cyclosporine, used to block *P*-glycoprotein-mediated efflux, and second generation ABC transporter inhibitors, such as PSC 833 and VX-710, have failed in cancer clinical trials (Dean et al., 2005). In addition, CSCs drug resistant have increased expression of repairing enzyme to chemical lesions made by reactive oxygen species and DNA damaging agents such as the topoisomerase inhibitors Etoposide, Adriamycin, and radiation therapy (Wang et al., 2015). In response to these agents, CSCs enter in a quiescent state, or senescence, and become insensitive to cell-cycle active chemotherapy (Campisi, 2013; Giuffrida et al., 2016). It is known that loss of PTEN induces senescence via a mechanism that requires p53. Survival factors released by cancer secretomes, and stress signal produced by damaged cells in tumor microenvironment, have key roles in cancer acquired resistance (Obenauf et al., 2015; Wang et al., 2015). Thus, to further dissection of these mechanisms we need to pursue new technical ways and use multiple target therapies to reverse senescence and kill proliferating CSCs.

Cancer stem cells exert multicellular functions in tumor tissue-specific networks and immune surveillance. More important, CSCs display differentiation-state plasticity that allow cancer cells to undergo epithelial to mesenchymal transition (EMT), which is a process that cancer cells gain migratory and invasive properties (Egeblad et al., 2010; Chaffer et al., 2011; Clevers, 2011; Gupta et al., 2011; Wang et al., 2015). The molecular profiling of CSCs are based on the expression of stem cell markers (CD34, CD44, CD133, ALDH, etc) and EMT gene drivers such as ETV5 (Ets Variant 5), NOTCH1, SNAI1 (Snail family zinc finger 1), TGFB1, among other genes (Chaffer and Weinberg, 2011; Clevers, 2011). Tumor-associated stromal cells and immune cells secrete soluble and insoluble glycoproteins in microenvironment that confer cell adhesion-mediated drug resistance (Obenauf et al., 2015; Wang et al., 2015). Some of these proteins may display potential biomarkers for targeted therapies (Egeblad, 2011; McGranahan and Swanton, 2015; Obenauf et al., 2015). The identification of microenvironmental changes that take place during tumor regression and resistance could be used for the design of more effective cancer treatment strategies (Pietras and Ostman, 2010; Almendro et al., 2014). Recent studies have used integrated proteogenomic approaches on tumor fragments to investigate intratumor heterogeneity and changes during chemotherapy in distinct cancer subtypes that favor cancer resistance and tumor evolution (Almendro et al., 2014; Mertins et al., 2016). Given the success of orthotopic implantation of human tumors in humanized mice models (Hidalgo et al., 2014; Shultz et al., 2014), we envisioned that future PDX studies will help us to understanding how conventional drugs drive selection of CSC clones within cancer population at different human-mouse tissue microenvironments. A significant promise is the use of multidimensional profiling of individual patient-CSC lines in *in vivo* models. Culture system of CTCs (circulating tumor cells) from individual patients is now being applied to test the drug susceptibility to diverse treatment regimens (Clevers, 2011; Almendro et al., 2014). In this context, several biological agents and small molecules targeting distinct components that control self-renewal and differentiation of CSCs, such as Notch, Hedgehog and WNT signaling pathway, are undergoing clinical trials (Zardavas et al., 2013). In this direction, K^+^ ionophore antibiotic salinomycin is one promising candidate under validation to CSC targeted therapy to triple-negative breast cancer patients (Gupta et al., 2009).

The validation of relevant compounds in clinical setting takes long time and typically results in enormous costs and failure rates. Co-clinical studies using matched cancer cell lines and PDX models are likely to have routine and successful applications that could bypass barriers to next generation cancer targeted gene therapies. Finally, oncogenomic portals and cancer informatics tools are ideal approaches for querying and retrieval of cancer’s gene biomarkers coupled with drug sensitivity and resistance. We anticipate that working in collaborative spirit, lab scientists, and clinical oncologists will ultimately find, design and validate novel and effective therapeutic strategies to improve personalized cancer medicine.

## Author Contributions

JB selected and reviewed the literature articles and wrote the manuscript. BS, MP-S, JN, and DM contributed to design and performed the experiments. All authors read and approved the manuscript.

## Conflict of Interest Statement

The authors declare that the research was conducted in the absence of any commercial or financial relationships that could be construed as a potential conflict of interest.

The reviewer VB and handling Editor declared their shared affiliation, and the handling Editor states that the process nevertheless met the standards of a fair and objective review.

## Footnotes

1 http://www.rcsb.org/pdb/home/home.do

2 www.pdbbind.org.cn

3 http://discover.nci.nih.gov/cellminer/

4 http://www.broadinstitute.org/ccle

5 http://www.broadinstitute.org/cmap

6 www.cancerRxgene.org

7 https://ocg.cancer.gov/programs/ctd2.

8 http://www.cbioportal.org

9 http://www.cytoscape.org/

10 http://discover.nci.nih.gov/cellminer/

11 http://wzw.tum.de/proteomics/nci60.

12 www.broadinstitute.org/cmap

13 http://www.lincsproject.org/.

14 https://www.broadinstitute.org/cmap/

15 http://www.ncbi.nlm.nih.gov/omim.
